# Aldosterone Synthase Deficiency Type I in a Neonate: Diagnostic, Genetic, and Therapeutic Insights From a Novel CYP11B2 Variant

**DOI:** 10.7759/cureus.97170

**Published:** 2025-11-18

**Authors:** Haydy M Khalifa, Rayan H Mohamed, Hisham Y Hassan, Fahad A Al-Qashar, Haya Alkhayyat

**Affiliations:** 1 Department of Pediatrics, Bahrain Defence Force Hospital, Royal Medical Services, Riffa, BHR; 2 Banoon Assisted Reproduction Technology (ART) and Cytogenetics Centre, Bahrain Defence Force Hospital, Royal Medical Services, Riffa, BHR

**Keywords:** aldosterone, aldosterone synthase deficiency, corticosterone 18-monooxygenase deficiency, corticosterone methyloxidase deficiency, cy11b2 gene

## Abstract

Aldosterone synthase deficiency (ASD), also known as corticosterone methyl oxidase deficiency, is a rare autosomal recessive disorder caused by inactivating mutations in the *CYP11B2* gene (Cytochrome P450 family 11 subfamily B member 2). ASD is classified into two subtypes: type I (Corticosterone methyl oxidase (CMO) I) and type II (CMO II), with type I characterized by minimal or absent aldosterone production and a more severe clinical phenotype. We report the case of a four-year-old male child who presented neonatally with respiratory distress, hyperkalemia, and hyponatremia. Comprehensive workup, including genetic analysis, confirmed CMO I deficiency due to a novel homozygous *CYP11B2* variant (*NM_000498.3:c.239+1G>A*), located at the donor splice site of intron 1. Early initiation of fludrocortisone and hydrocortisone led to favorable growth and development. This case underscores the importance of early recognition, genetic confirmation, and tailored therapy in managing rare neonatal electrolyte imbalances such as ASD type I.

## Introduction

Aldosterone is a vital steroid hormone synthesized in the zona glomerulosa of the adrenal cortex, playing a central role in electrolyte and fluid homeostasis. By promoting sodium reabsorption and potassium excretion in the distal renal tubules, aldosterone regulates blood pressure and extracellular fluid volume [1,2]. Its secretion is primarily governed by the renin-angiotensin-aldosterone system (RAAS), which is activated in response to hypovolemia, hypotension, or hyperkalemia [3].

Aldosterone biosynthesis is catalyzed by the mitochondrial enzyme aldosterone synthase, encoded by the *CYP11B2* gene (cytochrome P450 family 11 subfamily B member 2), located on chromosome 8q24.3 [4,5]. This enzyme mediates three sequential reactions-11β-hydroxylation of deoxycorticosterone to corticosterone, 18-hydroxylation, and 18-oxidation of 18-hydroxycorticosterone-ultimately producing aldosterone. The 11β-hydroxylation step is shared with *CYP11B1*, which encodes 11β-hydroxylase; however, 18-hydroxylation and 18-oxidation are unique to *CYP11B2*, accounting for the final steps of aldosterone formation. Deficiency of aldosterone synthase disrupts this tightly regulated pathway, leading to clinically significant mineralocorticoid deficiency [6].

Mutations in *CYP11B2* disrupt this biosynthetic pathway, resulting in isolated aldosterone synthase deficiency (ASD), a rare autosomal-recessive disorder [7,8]. ASD is classified into two subtypes based on the level of impairment in aldosterone biosynthesis: type I (corticosterone methyl oxidase (CMO) I) and type II (CMO II) [9,10]. Type I is characterized by a near-complete absence of aldosterone and low or undetectable 18-hydroxycorticosterone, leading to a more severe phenotype. In contrast, type II retains partial enzyme activity, resulting in detectable 18-hydroxycorticosterone and a milder clinical course [11,12].

Clinically, ASD often presents in the neonatal period or early infancy with salt-wasting crises, manifesting as hyponatremia, hyperkalemia, metabolic acidosis, dehydration, hypotension, and failure to thrive [7,8]. Severe cases may progress to seizures or coma if not promptly recognized and treated [9]. Unlike congenital adrenal hyperplasia, ASD does not cause virilization or hyperpigmentation, which helps distinguish it from other forms of adrenal insufficiency [4].

The diagnosis of ASD relies on a combination of clinical suspicion, biochemical evaluation (notably elevated plasma renin activity and low aldosterone), and molecular confirmation via genetic testing of the *CYP11B2* gene. Over forty pathogenic variants have been described, with variable genotype-phenotype correlations [6,10]. More than 200 *CYP11B2* variants have been identified to date, including single-nucleotide polymorphisms (SNPs) that affect enzyme activity and aldosterone synthesis [6,10]. Genetic confirmation is typically achieved using targeted Sanger sequencing or next-generation sequencing (NGS) panels targeting congenital adrenal hyperplasia-related genes for comprehensive variant detection. Early molecular confirmation is crucial for accurate diagnosis, management, and genetic counseling, particularly in populations with a high rate of consanguinity [11].

While ASD is extremely rare and its true prevalence remains undefined, clinicians should maintain a high index of suspicion in infants presenting with unexplained electrolyte disturbances and failure to thrive [8,10]. Early recognition and initiation of mineralocorticoid replacement therapy dramatically improve prognosis, allowing affected individuals to achieve normal growth and development [7].

This report presents the case of a neonate with ASD type I due to a novel homozygous *CYP11B2* variant, highlighting diagnostic challenges, the importance of genomic analysis, and the need for comprehensive family counseling and screening.

## Case presentation

**Figure 1:**
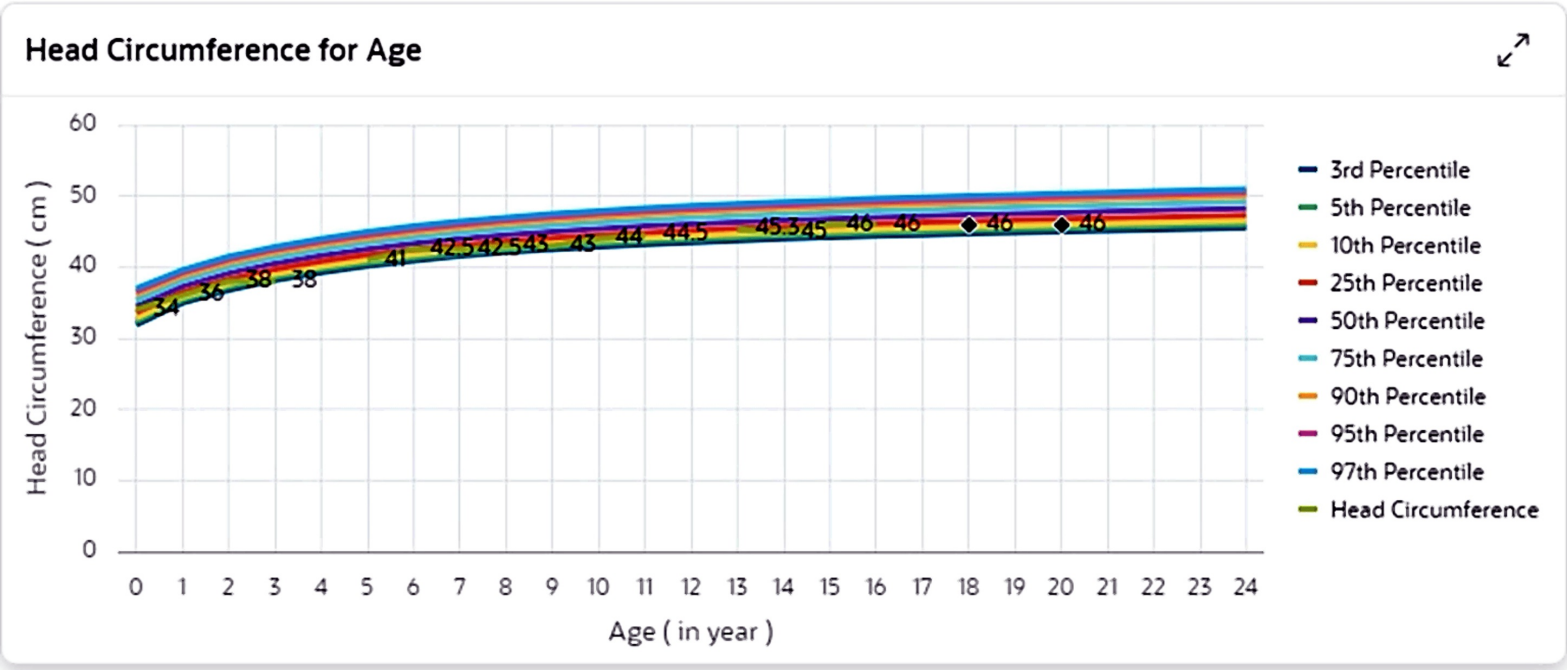
Growth tracking using WHO/CDC percentiles for head circumference — the child’s head circumference is within the normal range, consistently tracking between the 50th and 75th percentiles.

**Figure 2:**
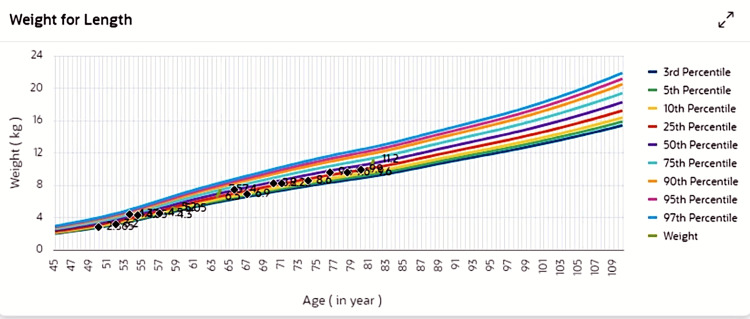
Growth tracking using WHO/CDC percentiles for weight — the child’s weight is within the normal range, consistently tracking between the 25th and 50th percentiles.

**Figure 3:**
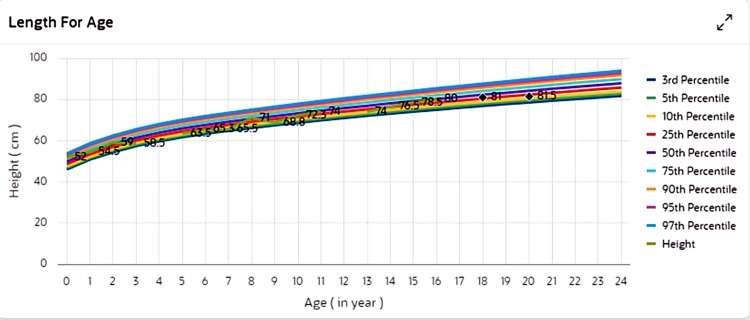
Growth tracking using WHO/CDC percentiles for length — the child’s length is within the normal range, consistently tracking between the 25th and 50th percentiles for age.

The patient is a four-year-old male child of Arab descent, born at term (38 weeks of gestation) via spontaneous vaginal delivery, with a birth weight appropriate for gestational age (Figures 1-3). There were no antenatal complications. However, the family history was notable for the neonatal death of a sibling due to an undetermined cause.

At birth, he was evaluated for tachypnea and electrolyte disturbances, prompting sepsis screening and metabolic workup. He was normotensive with normal male genitalia and bilateral descended testes. His systemic examination was unremarkable.

Initial investigations revealed hyponatremia and persistent hyperkalemia with normal renal function. Empiric antibiotic therapy with intravenous ampicillin and gentamicin was administered for seven days; blood cultures remained negative. Hyperkalemia was managed with IV calcium gluconate, salbutamol nebulizers, and insulin-glucose infusion, which normalized potassium levels.

Hormonal investigations demonstrated normal adrenocorticotropic hormone (ACTH) (23.46 ng/L; normal 10-60 pg/mL), normal cortisol (317.2 ng/mL; normal 90-690), low aldosterone (5.55 ng/dL; normal 5-90), and markedly elevated plasma renin activity (665.7 ng/mL/hour; normal 2-35). The 17-hydroxyprogesterone level was within the normal range (12.49 ng/mL; normal 1-28) (Table 1). Karyotype analysis confirmed a normal male pattern. He was initially started on hydrocortisone, fludrocortisone, and 3% sodium chloride. Following confirmation of isolated mineralocorticoid deficiency based on hormonal investigations, hydrocortisone was discontinued.

**Table 1:** Laboratory investigations, showing electrolyte imbalance and hormonal profile compared to normal reference ranges. ACTH: adrenocorticotropic hormone

Parameter	Patient Value	Reference Range
Sodium	126 mmol/L	135 – 145 mmol/L
Potassium	7.54 mmol/L	3.5 – 5 mmol/L
Cortisol	317.2 ng/mL	140 – 690 ng/mL
ACTH	23.46 ng/L	10–60 pg/mL
Aldosterone	5.55 ng/dL	5 – 90 ng/dL
Renin	665.7 ng/mL/hr	2 – 35 ng/mL/hr
Dehydroepiandrosterone	20.32 µg/dL	5 – 70 µg/dL
Testosterone	2.91 ng/mL	0.1 – 2.5 ng/mL
17-hydroxyprogesterone	12.49 ng/mL	1 – 28 ng/mL

Imaging studies, including renal ultrasonography, confirmed normal renal anatomy and echogenicity, with no evidence of hydronephrosis or structural abnormalities.

Genetic counseling was provided to the family, and solo whole-exome sequencing (WES) (CENTOGENE GmbH, Rostock, Germany) was performed at six months of age. This revealed a novel homozygous variant in the *CYP11B2* gene (NM_000498.3:c.239+1G>A), located at the donor splice site of intron 1. This variant is predicted to disrupt normal splicing, resulting in a loss of enzyme function. To date, this variant has not been reported in the literature or in the gnomAD population database. According to the American College of Medical Genetics and Genomics/Association for Molecular Pathology (ACMG/AMP) guidelines, it was classified as likely pathogenic. Both parents were confirmed to be heterozygous carriers. Sanger sequencing confirmed that both parents were heterozygous carriers of the variant.

Based on the clinical, biochemical, and genetic findings, a diagnosis of CMO-I deficiency was established. The patient was advised to continue oral fludrocortisone and sodium supplementation. He demonstrated rapid and sustained clinical improvement, with normalization of electrolyte levels, catch-up growth, and achievement of normal developmental milestones during follow-up. Family screening and genetic counseling were offered to identify potential carriers and to guide future reproductive planning.

## Discussion

CMO I deficiency, also known as ASD type I, is a rare autosomal recessive disorder caused by inactivating mutations in the *CYP11B2* gene, which encodes the aldosterone synthase enzyme responsible for the terminal steps of aldosterone biosynthesis [12,13]. This enzymatic defect results in markedly reduced or absent aldosterone production, while levels of its immediate precursor, 18-hydroxycorticosterone, remain low or within the normal range, leading to an elevated corticosterone-to-18-hydroxycorticosterone ratio characteristic of CMO I deficiency [14].

Clinically, ASD type I typically presents in early infancy with salt-wasting crises. Affected infants present with failure to thrive, recurrent vomiting, dehydration, hypotension, hyponatremia, hyperkalemia, and metabolic acidosis [15]. These derangements may progress to life-threatening complications such as seizures or coma if not promptly recognized and treated [16]. Because cortisol synthesis is preserved and androgen excess is absent, virilization and hyperpigmentation do not occur, findings that help distinguish ASD from adrenal insufficiency disorders such as classic congenital adrenal hyperplasia. In the present case, the neonatal onset of severe electrolyte derangements with normal cortisol levels and absence of androgenic signs supported a diagnosis of isolated mineralocorticoid deficiency [15,12].

The diagnosis of ASD type I relies on a combination of careful clinical assessment, targeted biochemical evaluation, and molecular confirmation. Biochemically, patients typically exhibit elevated plasma renin activity with undetectable or markedly reduced aldosterone levels, while cortisol and 17-hydroxyprogesterone concentrations remain normal, findings indicative of isolated hypoaldosteronism [13]. Measurement of precursor-to-product steroid ratios, such as 11-deoxycorticosterone to corticosterone or 18-hydroxycorticosterone to aldosterone, provides further diagnostic support by revealing an enzymatic block in the terminal steps of aldosterone synthesis [17]. Definitive genetic confirmation is achieved through WES, which enables the identification of pathogenic variants in the *CYP11B2* gene responsible for aldosterone synthase deficiency.

Definitive confirmation is achieved through molecular genetic analysis of* CYP11B2*, which reveals biallelic pathogenic variants [17]. In the present case, identification of a novel homozygous splice-site variant (NM_000498.3:c.239+1G>A) in *CYP11B2* confirmed the biochemical diagnosis. To date, more than 40 pathogenic variants of *CYP11B2* have been described, encompassing missense, nonsense, frameshift, and splice-site mutations, reflecting the wide genetic and phenotypic heterogeneity of ASD type I [10,18]. Variable expressivity and incomplete penetrance are well documented; for instance, individuals homozygous for the same mutation may exhibit markedly different clinical severity, as observed in one family where an asymptomatic parent and a critically ill neonate shared an identical variant [9,18].

While molecular testing is the gold standard for confirming ASD type I, it carries certain limitations, such as the potential inability to detect deep intronic or regulatory variants. Functional validation studies may further clarify the pathogenicity of splice-site changes like the one identified in this case. Genetic confirmation is therefore crucial not only for diagnostic accuracy but also for guiding management and genetic counseling. Early identification of the causative mutation allows for parental carrier testing, prenatal or newborn screening in subsequent pregnancies, and cascade screening of at-risk relatives [14].

Management of ASD type I is based on lifelong mineralocorticoid replacement with fludrocortisone, accompanied by liberal sodium supplementation to restore electrolyte balance and support normal growth and neurodevelopment. Early initiation of therapy is essential to prevent life-threatening salt-wasting episodes and optimize neurodevelopmental outcomes [14]. In the present patient, prompt treatment produced rapid clinical improvement, normalization of serum electrolytes, and catch-up growth, consistent with previous reports describing excellent response to fludrocortisone in this condition [13,15].

Family screening and genetic counseling are integral components of comprehensive management, particularly in populations with a high prevalence of consanguinity, such as the community described in this report [16]. Identification of heterozygous carriers enables informed reproductive decision-making and early recognition of affected offspring. Given the autosomal recessive inheritance pattern, each sibling of an affected individual has a 25% risk of being affected if both parents carry a pathogenic *CYP11B2* variant [19].

Despite its rarity, ASD type I should be considered in infants presenting with unexplained hyponatremia and hyperkalemia in the absence of virilization or hyperpigmentation [16]. The differential diagnosis includes other causes of hypoaldosteronism or mineralocorticoid resistance, such as pseudohypoaldosteronism type I and salt-wasting forms of congenital adrenal hyperplasia [10]. Genetic analysis remains indispensable for distinguishing among these disorders and establishing a definitive diagnosis. Long-term outcomes are generally favorable with adherence to therapy, although discontinuation of fludrocortisone or reduced salt intake may precipitate recurrence of symptoms, particularly during intercurrent illness or physiological stress [14]. Therefore, sustained follow-up and family education are essential to ensure lifelong compliance and early detection of breakthrough episodes.

In summary, ASD type I is a rare but critical endocrine disorder that must be recognized promptly in neonates with salt-wasting crises. Early biochemical evaluation and confirmatory genetic testing are pivotal for the timely initiation of therapy and comprehensive family-centered management [12]. With appropriate treatment and vigilant follow-up, affected infants can achieve normal growth and development, underscoring the need for awareness of this condition despite its rarity.

## Conclusions

ASD type I is a rare but potentially life-threatening disorder that should be considered in neonates presenting with unexplained hyponatremia, hyperkalemia, and metabolic acidosis, particularly in the absence of virilization or abnormal pigmentation. Early and accurate diagnosis is crucial, as prompt initiation of hormone replacement therapy with fludrocortisone and sodium supplementation can prevent severe complications and support normal growth and development.

Genetic confirmation via *CYP11B2* analysis not only establishes the diagnosis but also enables effective family counseling, cascade screening, and informed reproductive planning. In populations with high rates of consanguinity or a relevant family history, heightened clinical vigilance is warranted to facilitate early detection and intervention. With appropriate management, long-term prognosis is favorable, underscoring the importance of early recognition, comprehensive care, and ongoing follow-up for affected individuals and their families.

## References

[1] Funder JW (2012). Aldosterone and mineralocorticoid receptors: a personal reflection. Mol Cell Endocrinol.

[2] Lifton RP, Gharavi AG, Geller DS (2001). Molecular mechanisms of human hypertension. Cell.

[3] Connell JM, Davies E (2005). The new biology of aldosterone. J Endocrinol.

[4] Kayes-Wandover KM, White PC (2000). Steroidogenic enzyme gene expression in the human heart. J Clin Endocrinol Metab.

[5] White PC (2004). Aldosterone synthase deficiency and related disorders. Mol Cell Endocrinol.

[6] Portrat-Doyen S, Tourniaire J, Richard O (1998). Isolated aldosterone synthase deficiency caused by simultaneous E198D and V386A mutations in the CYP11B2 gene. J Clin Endocrinol Metab.

[7] Hattangady NG, Olala LO, Bollag WB, Rainey WE (2012). Acute and chronic regulation of aldosterone production. Mol Cell Endocrinol.

[8] Pascoe L, Curnow KM, Slutsker L, Rösler A, White PC (1992). Mutations in the human CYP11B2 (aldosterone synthase) gene causing corticosterone methyloxidase II deficiency. Proc Natl Acad Sci U S A.

[9] Nguyen HH, Hannemann F, Hartmann MF, Malunowicz EM, Wudy SA, Bernhardt R (2010). Five novel mutations in CYP11B2 gene detected in patients with aldosterone synthase deficiency type I: functional characterization and structural analyses. Mol Genet Metab.

[10] Miao H, Yu Z, Lu L (2019). Analysis of novel heterozygous mutations in the CYP11B2 gene causing congenital aldosterone synthase deficiency and literature review. Steroids.

[11] Gao X, Yamazaki Y, Tezuka Y (2021). Pathology of aldosterone biosynthesis and its action. Tohoku J Exp Med.

[12] Merakou C, Fylaktou I, Sertedaki A (2021). Molecular analysis of the CYP11B2 gene in 62 patients with hypoaldosteronism due to aldosterone synthase deficiency. J Clin Endocrinol Metab.

[13] Klomchan T, Supornsilchai V, Wacharasindhu S, Shotelersuk V, Sahakitrungruang T (2012). Novel CYP11B2 mutation causing aldosterone synthase (P450c11AS) deficiency. Eur J Pediatr.

[14] Hui E, Yeung MC, Cheung PT (2014). The clinical significance of aldosterone synthase deficiency: report of a novel mutation in the CYP11B2 gene. BMC Endocr Disord.

[15] Pela I, Capirchio L, Menchini C, Anzilotti G, Seminara S (2013). Isolated aldosterone deficiency in two infants: Mistakes and dilemmas in the diagnosis and treatment of a rare disease. Open J Ped.

[16] Faingelernt Y, Hershkovitz E, Abu-Libdeh B (2021). Aldosterone synthase (CYP11B2) deficiency among Palestinian infants: three novel variants and genetic heterogeneity. Am J Med Genet A.

[17] Løvås K, McFarlane I, Nguyen HH (2009). A novel CYP11B2 gene mutation in an Asian family with aldosterone synthase deficiency. J Clin Endocrinol Metab.

[18] Garrelfs MR, Rinne T, Hillebrand JJ (2024). Identification of a novel CYP11B2 variant in a family with varying degrees of aldosterone synthase deficiency. J Clin Res Pediatr Endocrinol.

[19] Cohn R, Scherer S, Hamosh A (2024). Thompson & Thompson Genetics and Genomics in Medicine. https://www.mea.elsevierhealth.com/thompson-thompson-genetics-and-genomics-in-medicine-9780323547628.html?srsltid=AfmBOor2_iR3OqReiakeeNE3dtlhVFlaBdGDo6a0cCG2E_dfu32T2JIl.

